# Enhanced preoperative planning in congenital polydactyly: superior assessment of MCP/MTP joint angular deformity with 3D-FS-FSPGR MRI compared to conventional radiography

**DOI:** 10.3389/fped.2025.1679008

**Published:** 2026-01-08

**Authors:** Jie Li, Quan Yun, Yingyu Jia, Jiangtao Long, Qianqian Wang, Yuankai Yang, Shuming Xu

**Affiliations:** 1 Department of Orthopedics, Shanxi Children’s Hospital, Shanxi Maternal and Child Health Hospital, Taiyuan, China; 2 Department of Pediatrics, Shanxi Medical University, Taiyuan, China; 3 Department of Imaging, Shanxi Children’s Hospital, Shanxi Maternal and Child Health Hospital, Taiyuan, China

**Keywords:** precision medicine, congenital polydactyly, MRI, conventional radiography, angular deformity

## Abstract

**Purpose:**

Precise quantification of angular deformity at the metacarpophalangeal (MCP) or metatarsophalangeal (MTP) joint is paramount in congenital polydactyly surgery. It dictates the surgical center point and informs the necessity for corrective osteotomy. Conventional radiography (x-ray), while standard, suffers from inherent limitations in visualizing cartilage and soft tissue, compromising surgical planning. This study evaluates the clinical feasibility and superiority of a three-dimensional fat suppression rapid phase shifting gradient echo (3D-FS-FSPGR) magnetic resonance imaging (MRI) sequence for overcoming these limitations and achieving accurate preoperative angular assessment.

**Methods:**

Pediatric patients presenting with congenital polydactyly of the hands or feet underwent preoperative imaging with both standard x-ray and the 3D-FS-FSPGR MRI sequence. Evaluation focused on characterizing angular deformities at the affected joints. Direct comparative analysis assessed the visualization of osseous alignment, cartilage architecture, joint bifurcation planes, and surrounding soft tissues, alongside the accuracy of angular measurements derived from each modality.

**Results:**

Compared to x-ray, the 3D-FS-FSPGR MRI sequence demonstrated superior visualization of cartilage morphology, joint capsule anatomy, soft tissue, and articular surfaces at the MCP/MTP joints. This anatomical delineation translated to more accurate and reliable quantification of angular deformities. Crucially, significant discrepancies in measured joint angles were observed between MRI and x-ray. These differences are pronounced in cases where the morphology of the cartilage affects joint alignment and the complex branching planes.

**Conclusion:**

Conventional x-ray assessment in congenital polydactyly is limited in visualizing soft tissue and cartilage structures. The 3D-FS-FSPGR MRI sequence offers superior characterization of cartilaginous and soft tissue components, enabling more precise measurement of joint angular deformities. Its integration into the preoperative evaluation protocol demonstrates substantial clinical feasibility and tangible potential to optimize surgical site and reduce the incidence of postoperative deformities and functional impairment, thereby improving the long-term functional and aesthetic outcomes for these children.

## 1 Introduction

Congenital polydactyly represents a common congenital anomaly affecting the hands and feet (1, 2). While the precise pathogenesis remains incompletely elucidated, genetic factors are strongly implicated (3–5). Surgical reconstruction is universally recognized as the primary and most effective treatment modality (6, 7). Within polydactyly surgery, the angular deformity at the metacarpophalangeal (MCP) or metatarsophalangeal (MTP) joint is a critical determinant, guiding the selection of the surgical center point and the pivotal decision regarding the necessity of corrective osteotomy. Accurate preoperative measurement of this angle is therefore non-negotiable for effective surgical planning and the crucial prevention of postoperative malalignment and functional deficits as well.

Conventional radiography (x-ray) remains the most widely utilized preoperative imaging modality in pediatric polydactyly. It provides essential information on osseous angular deformity, joint bifurcation, and digital axial alignment, aiding in the determination of surgical center point. However, a fundamental and well-recognized limitation of x-ray is its inability to clearly delineate articular cartilage and soft tissue structures (8, 9). This inherent deficiency inevitably introduces measurement errors in assessing the accurate MCP or MTP joint deviation angle. Such inaccuracies can lead to the misidentification of the optimal surgical center point, subsequently contributing to postoperative angular deformities and compromised functional recovery.

Magnetic resonance imaging (MRI), with its exquisite visualization of cartilage and soft tissue anatomy, offers a compelling solution. This capability addresses the shortcomings of x-ray. This study specifically employed the three-dimensional fat suppression rapid phase shifting gradient echo (3D-FS-FSPGR) sequence to image the deformed digits in children with congenital polydactyly. By conducting a comparison with conventional x-ray, we aimed to evaluate the clinical feasibility and diagnostic superiority of MRI technique for the precise assessment of angular deformities at the MCP or MTP joints, thereby providing a more robust foundation for optimized surgical intervention.

## 2 Materials and methods

This study received approval from the Ethics Committee of Shanxi Children's Hospital (IRB-KYYN-2021-003), and informed consent was obtained from the children's parents or guardians.

### 2.1 Patient selection

A retrospective analysis was conducted on 55 children with congenital polydactyly (65 digits) who underwent surgical treatment at our hospital between May 2021 and April 2022. Inclusion criteria: (1) Diagnosis of congenital polydactyly with associated angular deformity; (2) Complete medical records; (3) First-time surgical treatment at our hospital, with adherence to prescribed rehabilitation exercises and regular follow-up attendance; (4) Absence of other congenital anomalies, genetic metabolic disorders, or neuromuscular diseases. Exclusion criteria: (1) Inability to cooperate with or tolerate contrast-enhanced MRI; (2) Non-standard preoperative x-ray positioning (standard anteroposterior or oblique views for thumbs); (3) Poor-quality MRI images.

### 2.2 Imaging protocol

#### 2.2.1 X-ray examination

All children underwent standard anteroposterior and lateral x-ray imaging of the affected hand before surgery. The DR system was used, with parameters adjusted according to the child's age and body size. Specific exposure parameters (kVp, mAs) were individually adjusted based on the child's age, body size, and tissue thickness to ensure image quality.

#### 2.2.2 MRI examination

All metallic objects were removed from the child and accompanying adults. For infants and young children unable to cooperate with the scan duration, appropriate sedation such as oral 10% chloral hydrate solution (0.5 mL/kg) or intramuscular sodium phenobarbital (5 mg/kg) was administered under pediatrician supervision. Imaging commenced only after confirmed sleep onset. All scans were performed on a GE Discovery 750 W 3.0 T MRI scanner. Axial, coronal, and sagittal T2-weighted (T2WI) and T1-weighted (T1WI) sequences were acquired. The key sequence for cartilage or soft tissue evaluation was performed with optimized parameters: TR 13.1 ms, TE 2.3 ms, FOV 20 cm, slice/gap 1.5 mm/−0.8 mm ov, matrix 320 × 224, NEX 3, FA 15°. Average scan time was ∼4 min. Fat-suppressed contrast-enhanced T1-weighted images cover from the distal forearm to the fingertips or leg to the toe tips.

Hands: Fix the hard cardboard on the palm, extend the fingers straight with the palm facing down, apply an 8-channel soft coil over the child's hand, and then secure it with sandbags. Position the child's body towards the healthy side, ensuring that the fingers on the affected side are as close as possible to the center of the coil and the main magnet to ensure image quality. The child should be in a supine position, with the head leading during the scan.

Feet: Fix the rigid cardboard to the sole of the foot, ensuring that the foot is perpendicular to the scanning bed. Use an 8-channel phased array dedicated coil for the ankle joint while the child is in a supine position, and scan with the foot in an advanced position.

### 2.3 Image post-processing

Perform maximum density projection reconstruction on the original images of the coronal 3D-FS-FSPGR sequence, adjusting the thickness of different layers to display the tendons, soft tissues, and blood vessels.

### 2.4 Image analysis

Two senior radiologists (attending physician level or higher) independently analyzed the x-ray and MRI 3D-FS-FSPGR images using double-blind method. The presence of angular deformity at the MCP/MTP joint was identified based on cartilage morphology. Subsequently, two senior orthopedic surgeons (attending physician level or higher) compared the angular measurements derived from x-rays and MRI. Discrepancies were resolved by consultation with a third, more senior physician to reach a consensus.

### 2.5 Surgical methods

The appropriate surgical approach was selected based on the Wassel classification, with intraoperative open exploration as the gold standard, and the accuracy of preoperative imaging classification was statistically analyzed.

## 3 Results

### 3.1 Patient characteristics

The study included 55 children (Median age: 11 months, IQR: 24) with congenital polydactyly (including toes). Unilateral deformities were present in 45 cases (81.8%), and bilateral deformities in 10 cases (18.2%). There were 26 girls (47.3%) and 29 boys (52.7%). According to the Wessel classification, there were 11 cases of Wessel type II, 16 of type III, 20 of type IV, 4 of type V, 5 of type VI, and 9 of type VII. Cases with angulation deformity were primarily concentrated in Wessel types III, IV, and V, for comparison (Table 1). Compared with actual intraoperative observation, x-rays can misjudge angulation and the selection of the surgical center, while MRI allows for accurate preoperative assessment (Table 2).

**Table 1:** Improved Tada scoring system.

Criterion score	Points
Range of motion (ROM)	
>70°	2
70–50°	1
<50°	0
Instability	
Negative	1
Positive	0
Malalignment	
<10°	2
10–20°	1
>20°	0
Subjective family opinion	
Acceptable function and cosmetic results	2
Acceptable functional or cosmetic result	1
Unacceptable functional and cosmetic results	0

**Table 2:** Phelps' postoperative evaluation methods for polydactyly surgery.

Criterion score	Excellent	Good	Poor
Pain	None	Occasional	Persistent
Difficulty in Wearing Shoes	None	Occasional	Frequent
Calluses	None	Painless Callus	Persistent Painful Callus
Preserved Little Toe Deformity	None	Mild	Significant
Scarring Satisfaction	Satisfied	Average	Unsatisfied

### 3.2 Intraoperative and postoperative findings

The MRI 3D-FS-FSPGR sequence clearly delineated the levels of cartilage bifurcation and bone tissue development, which were consistent with intraoperative observations. At the one-year postoperative follow-up, the reconstructed fingers exhibited significant improvement in shape, with normal bone alignment and no deviations, normal appearance of the nails, and no apparent scar contractures or hyperplasia. Finger extension, flexion, grasp, and opposition functions were performing well, with no postoperative deformities or recurrences observed. According to the improved Tada scoring system (Table 3) and Phelps scoring system (Table 4), the postoperative success rate was 87.7% (57/65).

**Table 3:** Summary of Wessel classification for cases.

Total	Wassel	
65	II	11
	III	16
	IV	20
	V	4
	VI	5
	VII	9

**Table 4:** Comparative analysis of intraoperative exploration, x-ray, and MRI results, with statistical evaluation of x-ray's misjudgment rate for actual multi-finger angular deformity and MRI's accuracy rate.

Wassel	Angularity detected in operation	X-ray misjudgment (%)	MRI accuracy (%)
III	16	10 (62.5)	10 (100.0)
IV	20	10 (50.0)	26 (100.0)
V	4	1 (25.0)	4 (100.0)

### 3.3 A comparative demonstration of polydactyly imaging

**Figure 1:**
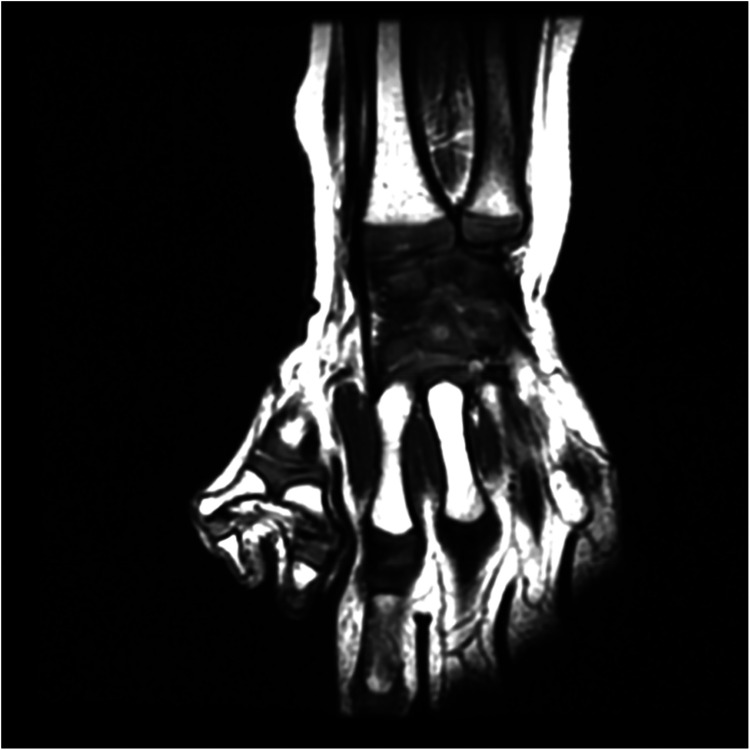
Preoperative MRI showing the angular deformity. The MRI provides detailed visualization of the cartilaginous structures and soft tissues.

**Figure 2:**
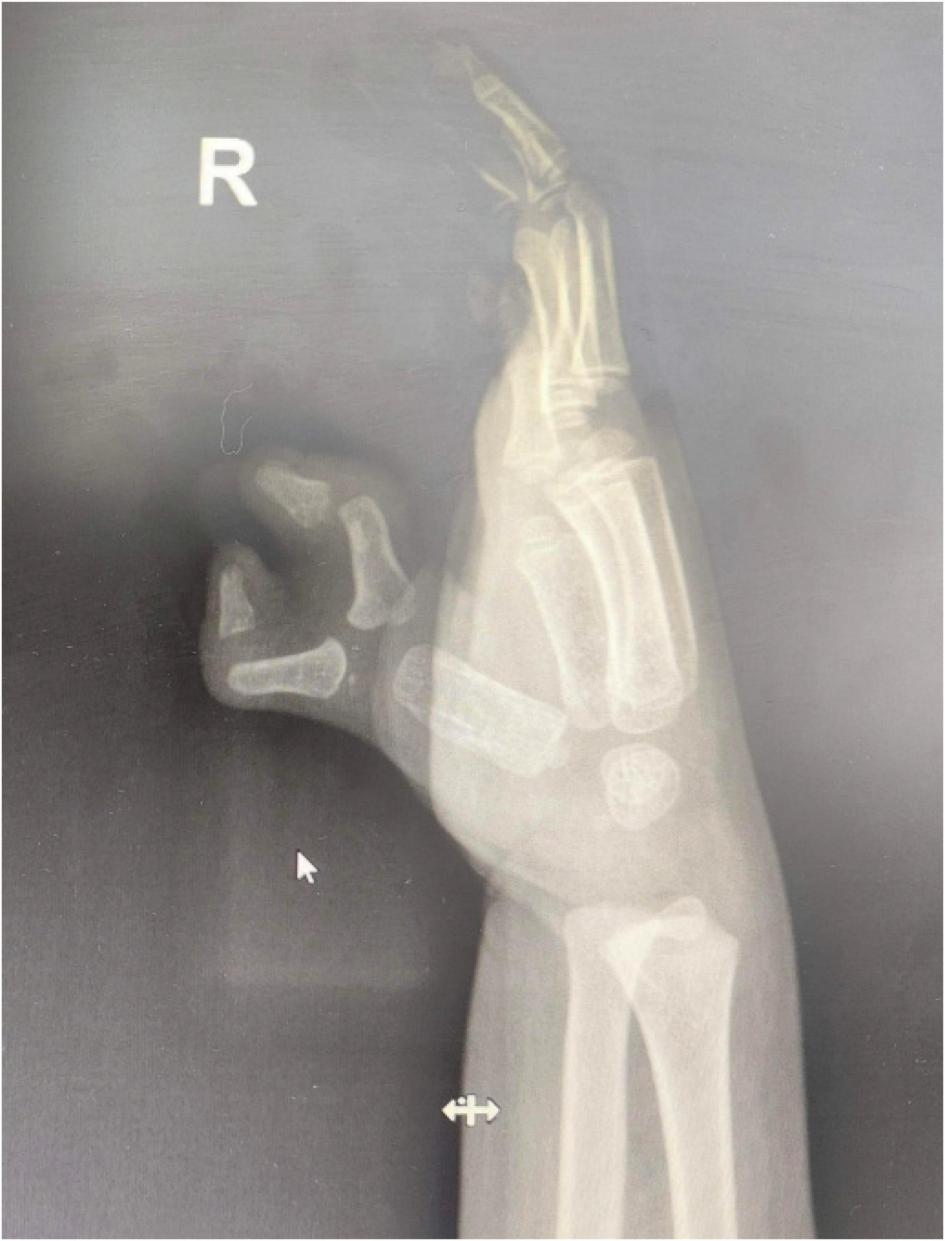
Preoperative x-ray showing the angular deformity. The standing x-ray image reveals the bony architecture under gravitational load.

**Figure 3:**
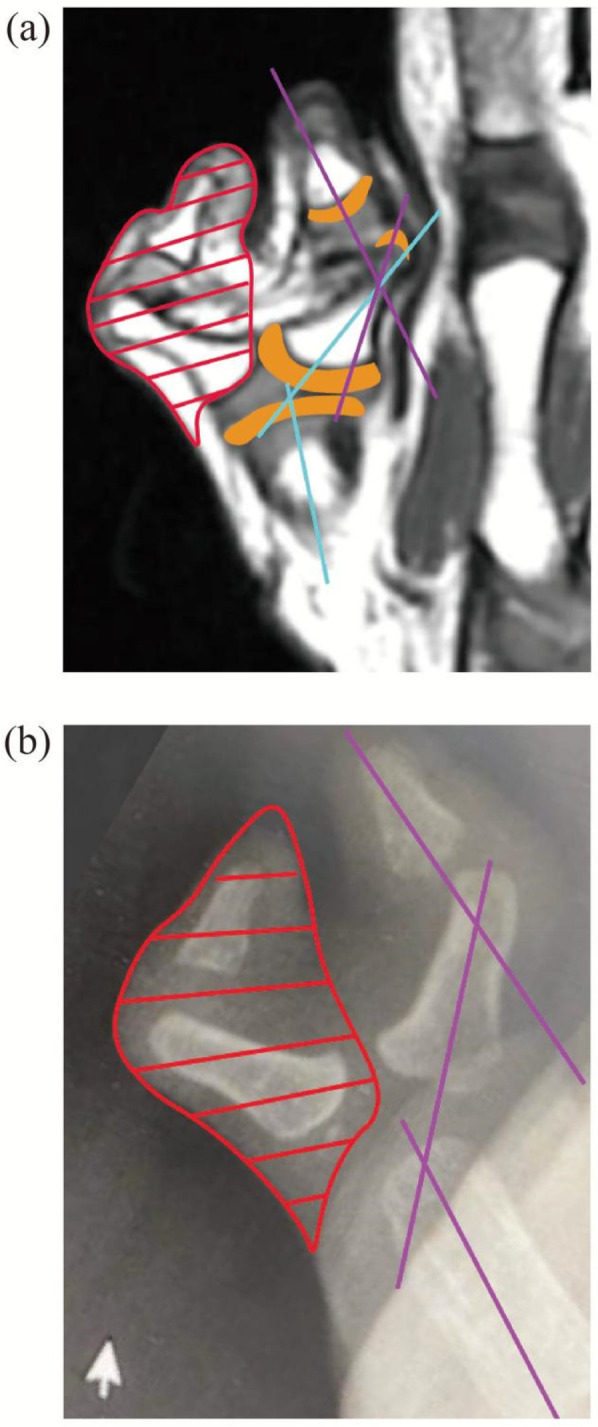
The osteotomy schematic diagram on both the x-ray and MRI images. **(a)** Preoperative planning conducted on the MRI image. The red shaded area indicates the portion to be resected, the orange-yellow line represents cartilage, the purple line indicates the mechanical axis between the two phalanges, and the blue line denotes the mechanical axis between the phalanx and metacarpal bone. **(b)** Preoperative planning conducted on the x-ray image. The red shaded area marks the section to be excised, and the perple line indicates the mechanical axis.

**Figure 4:**
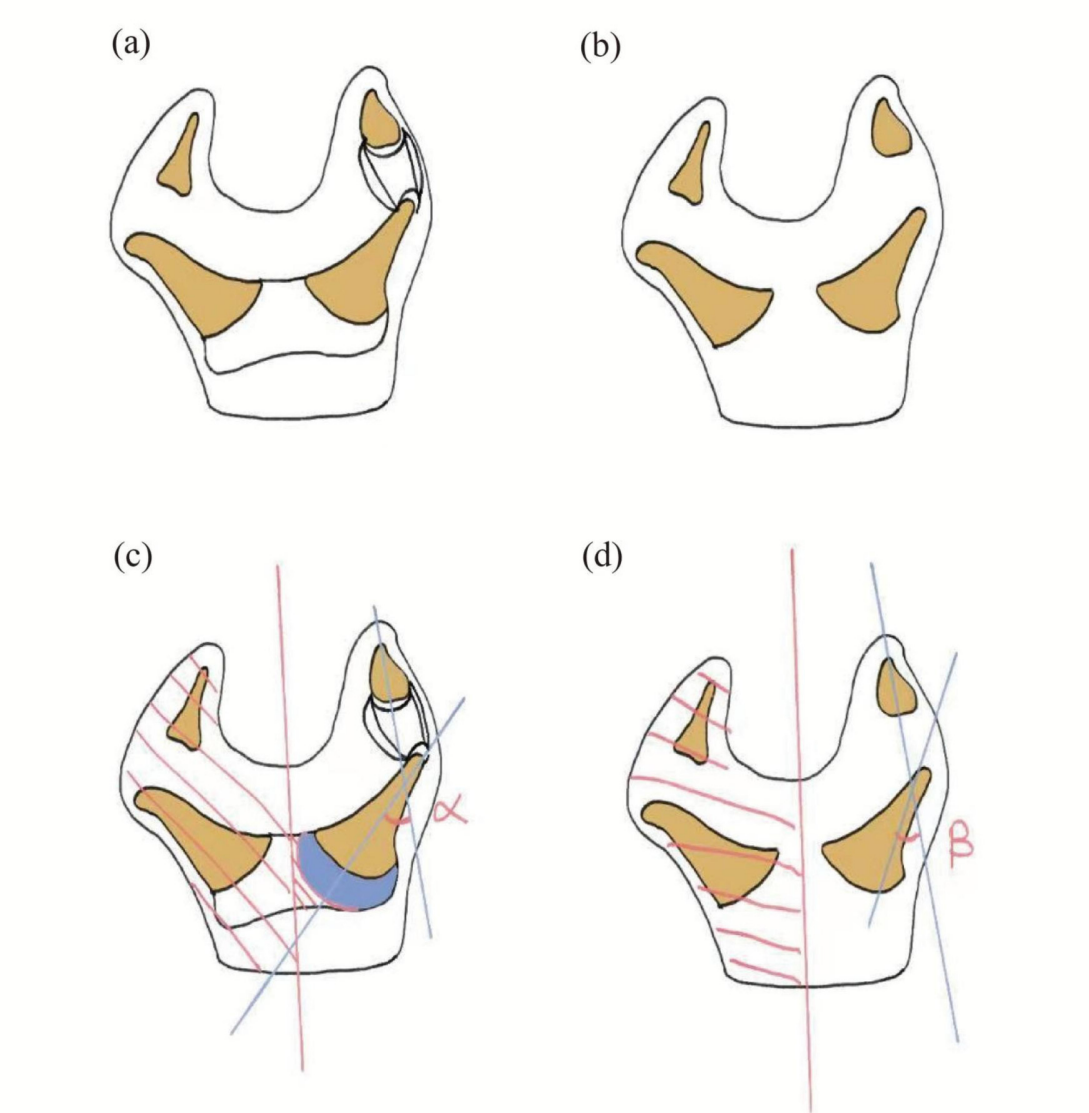
Schematic diagrams illustrating the angular deformities and measurement methods. **(a)** Schematic representations of the deformed anatomy based on MRI. **(b)** Schematic representations of the deformed anatomy based on x-ray. **(c)** Measurement of the α-angle based on the MRI, which incorporates the cartilaginous structures to determine the true mechanical axis and the correct surgical center of rotation. **(d)** Measurement of the β-angle based on the x-ray, where the absence of cartilage leads to a discrepancy in the perceived center of rotation compared to the MRI-based α-angle.

Both MRI and x-ray indicate the presence of angular deformity (Figures 1–3), with schematic diagrams shown in Figures 3a,b, 4a,b. Using professional electronic measurement tools, the angle of the deformity was measured and compared to determine the angle and surgical plan, accompanied by schematic diagrams (Figures 3, 4). The α angle represents the angular deformity in MRI (Figure 4c), while the β angle corresponds to the angular deformity in x-ray (Figure 4d). It is clearly observed that the discrepancy between the β angle and α angle is due to the absence of cartilage structure in the x-ray, resulting in a significant deviation in the positioning of the surgical center point. The surgery was performed based on the α angle, including osteotomy and soft tissue balancing.

### 3.4 Overall postoperative outcome evaluation

**Figure 5:**
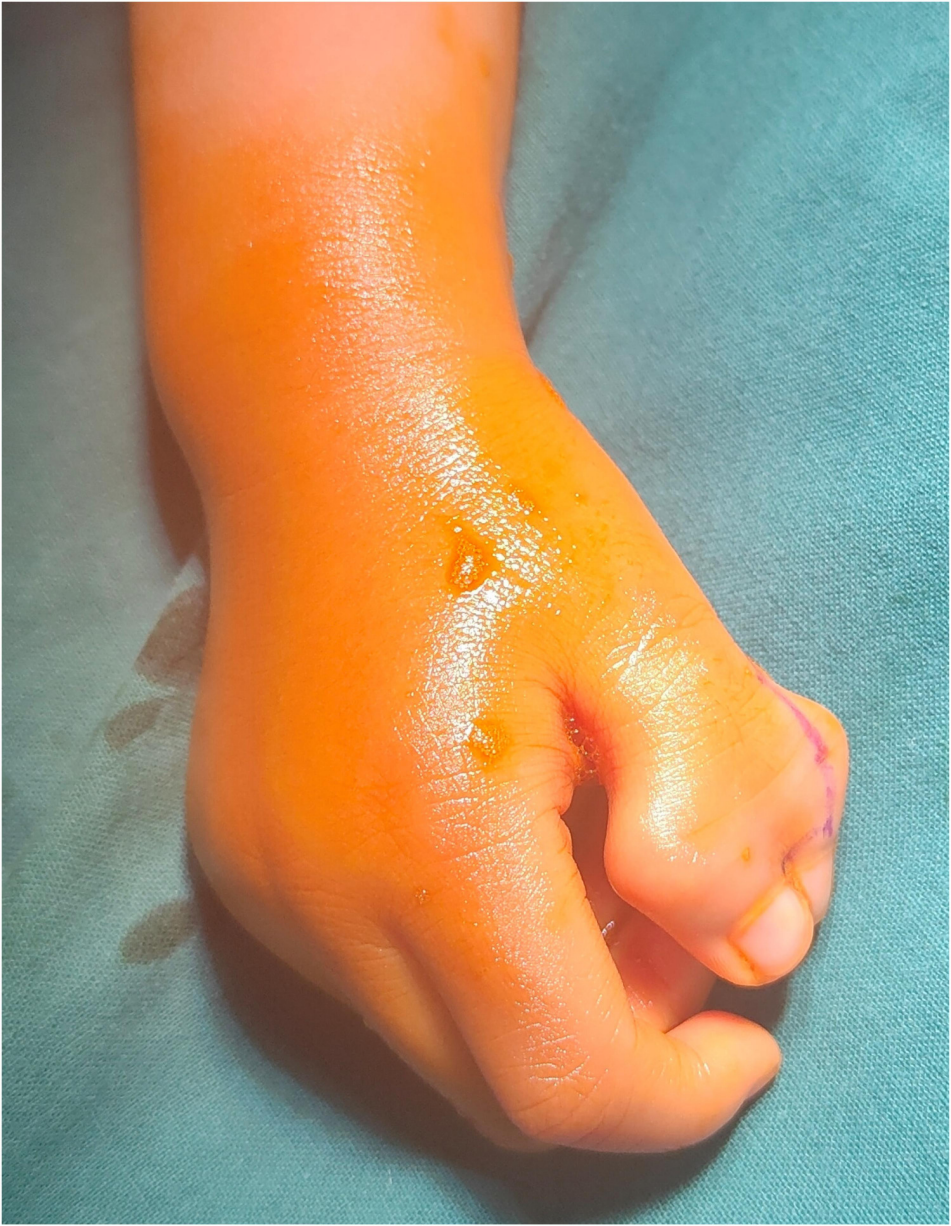
Preoperative photo. The preoperative clinical photograph of a pediatric patient with Wassel Type III polydactyly are shown.

**Figure 6:**
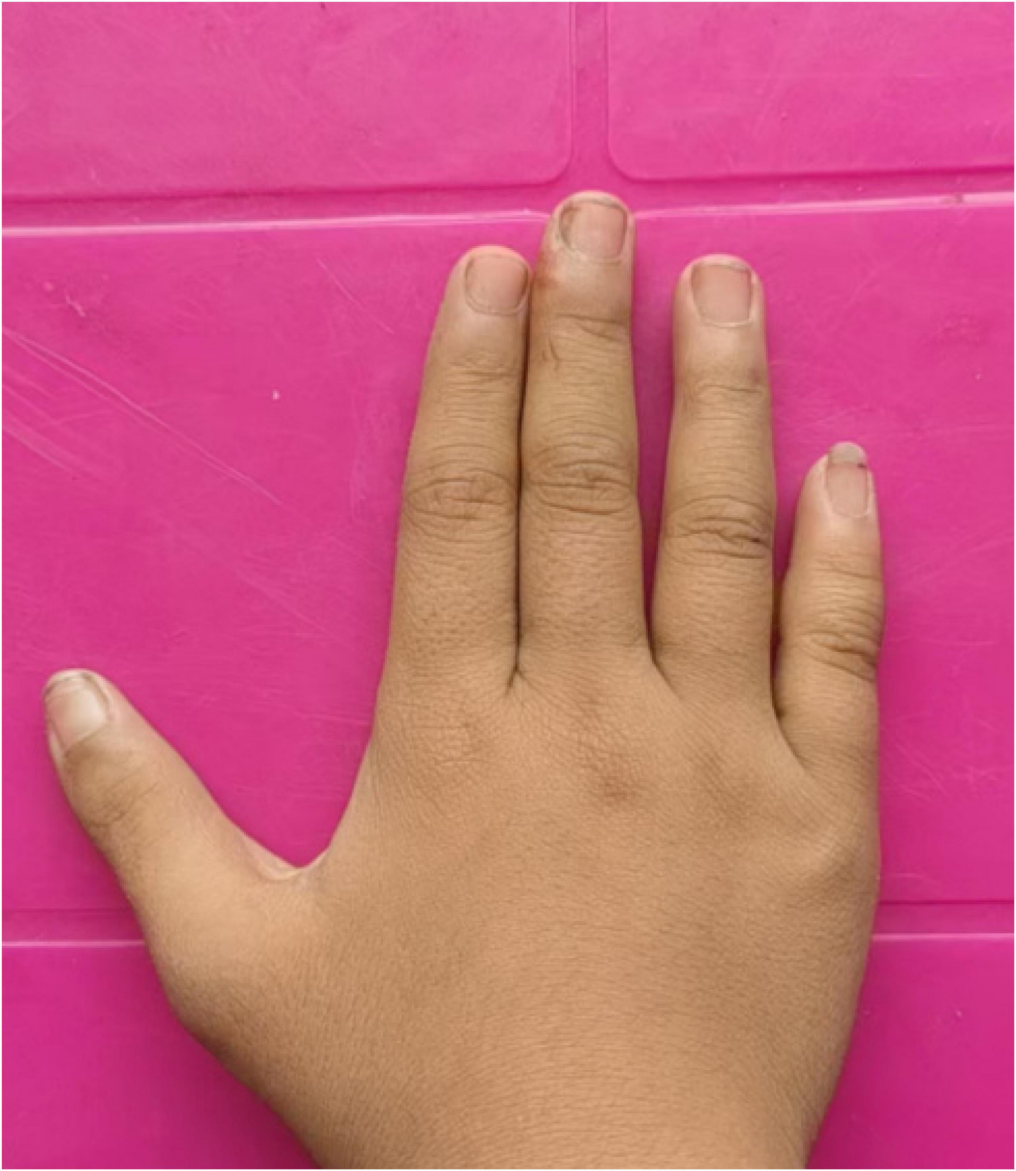
Postoperative follow-up assessment at one year. The photo demonstrates the satisfactory aesthetic appearance of the reconstructed thumb, with a well-healed incision, normal nail appearance, and absence of significant scarring or deviation.

All patients underwent successful surgery, with primary incision healing and no complications such as infection or skin necrosis. One-year follow-up showed significant improvement in the appearance of the reconstructed thumbs, with normal bone axis restoration, no deviation of the digits, and normal nail appearance. There was no significant scarring or contracture. Thumb extension, flexion, grasping, and opposition functions were good, with no postoperative deformity or recurrence. Compared to the preoperative condition (Figure 5), the patients and their parents were satisfied with their postoperative recovery, and their daily activities and activities were satisfactory (Figure 6).

## 4 Discussion

Fifty-five children with congenital polydactyly who underwent surgery at our institution between May 2021 and April 2022 were selected for this study for three primary reasons. First, to ensure protocol homogeneity. This period marked the initial implementation of our institution's prospective standardized imaging protocol. Limiting the cohort to this time period ensured that all enrolled patients underwent identical preoperative examinations (including x-rays and 3D-FS-FSPGR MRI) and surgical planning procedures. This design rigorously controlled for temporal variability that may occur in clinical practice, a key advantage for feasibility studies. Second, the sample size was sufficient. Congenital polydactyly is a common condition at our institution. The enrollment of 55 patients (65 fingers) over a one-year period was a robust sample size, matching or exceeding the sample sizes of other pivotal studies in this field or similar studies employing novel surgical procedures or methods, demonstrating that this time period was sufficient to obtain meaningful data (9–19). Third, the study was aligned with the objectives. The core objective of this study was to conduct a paired comparison of the diagnostic performance of a novel MRI sequence with that of conventional x-rays. The one-year continuous enrollment period was sufficient to achieve this objective and provide strong preliminary evidence for the superiority of this technology. Some imaging studies, due to limited sample sizes in the short term, select cases spanning more than a year or several years (13, 14, 16, 18, 20). Other imaging studies, however, select and analyze data collected within just six months (21). Therefore, the timeframe for data selection is relatively flexible in innovative imaging research. A sufficient, illustrative sample size and the insights revealed by the cases themselves are more persuasive and worthy of attention.

Current research on osteotomy techniques for polydactyly primarily focuses on several procedures, including the On-top Osteotomy, Wedge Osteotomy, and Metatarsal Neck Osteotomy (22–28). The On-top Osteotomy is indicated for Wassel type VI radial polydactyly (thumb duplication with a triphalangeal component) or deformities at the metatarsal level in the foot. This procedure involves resecting the distal one-third of the metacarpal from the radial duplicate while preserving the proximal two-thirds of the metacarpal from the ulnar duplicate. The distal bony segment of the ulnar thumb is then transplanted to the base of the radial metacarpal to achieve bony fusion and realignment (22–24). A key advantage of this technique is its ability to preserve functional joints (such as the metacarpophalangeal joint or interphalangeal joint), thereby helping to avoid joint instability or residual valgus/varus deformities (22, 24). The Wedge Osteotomy is commonly used for realignment at the phalangeal or metatarsal level (25–27). It typically requires internal fixation with Kirschner wires (K-wires) to maintain the corrected position (25). This technique involves removing a wedge-shaped bone segment and directly closing the osteotomy site, which is effective for correcting angular deformities such as finger or toe deviation exceeding 15 degrees (26). The Metatarsal Neck Osteotomy is primarily used for postaxial polydactyly of the foot (between the 4th and 5th metatarsals) (28). It transfers the distally aligned toe to the preserved metatarsal via osteotomy, concurrently excising the supernumerary digit, narrowing the forefoot width, and restoring the alignment of the fifth toe. A critical consideration in performing these osteotomies is achieving soft tissue balance (22). Surgeons must reconstruct ligaments (e.g., the radial collateral ligament), tendons (e.g., the abductor pollicis brevis), and the joint capsule after osteotomy to ensure joint stability and dynamic function. Preoperative MRI plays a valuable role in planning by providing detailed assessment of these soft tissue structures.

The surgical treatment of congenital polydactyly with its complexity goes far beyond mere excision. Optimal functional and aesthetic reconstruction necessitates a strategic approach to managing redundant structures. From the perspective of bone correction, significant angular deformities invariably require concomitant osteotomy correction to achieve stable and aligned joints (8, 11, 29–31). Therefore, accurate measurement of angular deformities is particularly crucial. However, clinical practice has shown that x-rays are prone to misjudging angles (8). There are three main reasons: First, x-rays rely on bony landmarks to indirectly estimate joint angles, but cartilage abnormalities (such as spiny protrusions on the head of the metacarpal bone or uneven articular surfaces) can alter actual force line directions. Second, positioning difficulties in children often result in camera angle deviations, which further amplify measurement errors. Additionally, relying solely on the differences in the x-ray images of the thumb in cases of duplicate thumb deformity may influence the surgical approach. For example, some Wassel I and II type duplicate thumbs appear as widened proximal phalanges in the anteroposterior x-ray but do not show significant widening in the oblique view. Therefore, it is not possible to accurately determine the surgical plan based solely on the appearance of the x-ray, as the surgeon may overlook the enlargement of the phalanx, which could result in the affected thumb being thicker than the normal side post-operation, thereby impacting aesthetics.

Currently, there are various imaging assessments for measuring the angles of polydactyly, each with its own limitations in assisting diagnosis and treatment. For example, the three-dimensional CT scan can reveal detailed structures of the accessory bones and joints, aiding in the determination of force lines and angles; however, it inevitably exposes the child to radiation. Joint imaging can demonstrate the shape and position of joints, assess the condition of cartilage, and measure axes, but it is an invasive procedure, making it difficult to allocate sufficient time for preoperative planning. Ultrasound can examine the soft tissue, cartilage, and joints of the extra digits, but it is challenging to perform on children and lacks widespread applicability, with the possibility of false assessments. Although the x-ray are the most commonly used imaging technique for preoperative evaluation of polydactyly, its drawback of being unable to clearly distinguish soft tissues can't be neglected. In contrast, MRI is radiation-free and more readily applicable for children, offering better resolution for soft tissues, blood vessels, and nerves. To fully illustrate the preoperative value of MRI in treating children with polydactyly, this study primarily explores and compares the differences between x-ray and MRI in determining the center point for osteotomy surgery, concluding that MRI can effectively and accurately identify the osteotomy center point for polydactyly surgeries.

In order to better correct the force lines and achieve the ideal joint mobility, we compared x-ray images with MRI imaging. We clearly marked the positions of each structure and measured the angles of the metacarpophalangeal (metatarsophalangeal) joints to determine whether osteotomy or other surgical options are necessary. The comparison illustrates the disadvantages of solely using x-ray assessments and the benefits of incorporating MRI (Figures 1–4). The one-year postoperative follow-up results (Figure 6) indicate that the bone axis has returned to normal, functional recovery is satisfactory, and there are no postoperative secondary deformities.

The benefits of precise preoperative planning based on 3D-FS-FSPGR MRI go beyond simple functional recovery and extend to the level of plastic aesthetics and the long-term psychosocial development of children. Congenital hand deformities have a profound psychological impact on children and may cause problems such as social avoidance and damaged self-esteem (32). Through the precise definition of anatomical structures by MRI, the surgeon can achieve bone alignment and soft tissue balance that is closer to the physiological form. This normalization of appearance is crucial to alleviating the anxiety and inferiority that children may have due to physical differences and helping them to confidently integrate into society. It also greatly alleviates the psychological burden on parents (32–34).

It can be contributed to three main reasons: First, the preoperative evaluation plan was comprehensive, incorporating MRI along with x-ray images to repeatedly compare and assess the epiphyseal structure, accurately determining the angulation and locating the osteotomy point; second, to achieve reconstruction of both appearance and function, osteotomy surgery often requires tendon and other soft tissue balancing procedures. We utilized the clearly delineated bony and soft tissue structures provided by MRI to reduce potential risks of additional intraoperative damage; third, MRI can optimize the functional reconstruction plan by analyzing tendons (Figures 3, 4) and assessing postoperative joint mobility. Therefore, the surgical strategy clarified the focal point of the operation by clearly delineating the cartilage, tendons, and other soft tissue structures preoperatively.

However, this study has limitations. First, there is currently a lack of unified standards for angulation measurements and osteotomy angles, which necessitates the establishment of multi-center researches in the future. Second, the follow-up period for this group is limited, and further investigation is needed to determine whether the joint mobility of the metacarpophalangeal (metatarsophalangeal) joints will be further restricted as the children grow and develop. Additionally, the cost of MRI examinations is relatively high, and young children require sedation for cooperation, making the process somewhat complicated; future efforts will focus on exploring more convenient and efficient scanning sequences to enhance examination efficiency. Subsequent studies may also use standardized psychological assessment tools and quality of life scales to quantify the positive effects of surgical intervention on children's psychological adaptation and family well-being.

In conclusion, our research indicates that 3D-FS-FSPGR MRI provides an excellent and clinically feasible visualization solution for the joint angle deformities of the MCP or MTP in children with congenital polydactyly. It overcomes the limitations of traditional x-ray images by depicting the critical cartilage and soft tissue components of polydactyly. This significantly reduces the surgeon's reliance on intraoperative findings, contributing to the realization of precision medicine. Integrating this MRI sequence into the preoperative examination process can optimize the surgical site, objectively determine the osteotomy points, and ultimately minimize the risks of postoperative deformities and functional impairments, thereby improving the prognosis for our young patients.

## Data Availability

The datasets generated or analyzed during the study are not publicly available due to the protection of patients' privacy but are available from the corresponding author on reasonable request.
